# Interactions between Identity and Emotional Expression in Face Processing across the Lifespan: Evidence from Redundancy Gains

**DOI:** 10.1155/2014/136073

**Published:** 2014-04-15

**Authors:** Alla Yankouskaya, Pia Rotshtein, Glyn W. Humphreys

**Affiliations:** ^1^School of Psychology, University of Birmingham, Birmingham B15 2TT, UK; ^2^Department of Experimental Psychology, University of Oxford, Oxford OX1 3UD, UK

## Abstract

We tested how aging affects the integration of visual information from faces. Three groups of participants aged 20–30, 40–50, and 60–70 performed a divided attention task in which they had to detect the presence of a target facial identity or a target facial expression. Three target stimuli were used: (1) with the target identity but not the target expression, (2) with the target expression but not the target identity, and (3) with both the target identity and target expression (the redundant target condition). On nontarget trials the faces contained neither the target identity nor expression. All groups were faster in responding to a face containing both the target identity and emotion compared to faces containing either single target. Furthermore the redundancy gains for combined targets exceeded performance limits predicted by the independent processing of facial identity and emotion. These results are held across the age range. The results suggest that there is interactive processing of facial identity and emotion which is independent of the effects of cognitive aging. Older participants demonstrated reliably larger size of the redundancy gains compared to the young group that reflect a greater experience with faces. Alternative explanations are discussed.

## 1. Introduction

How does the ability to process information from faces change as we age? Prior work indicates that there are effects of aging on a number of aspects of face processing. For example, there is an age-related decline in recognition memory for faces [1–6], an effect that grows as the retention interval lengthens [7, 8] and memory load increases [9, 10].

In other cases though the effects of aging impact perceptual rather than memory processes [11–15]. For example, Obermeyer et al. [14] examined the effect of age on early perceptual stages of face processing by applying horizontal and vertical filtering to face images in a face recognition task. The results indicated a detrimental effect of age on the processing of horizontal information. Daniel and Bentin [12] examined the ability of people aged 70 to 90 years old to apply global, configural, and featural processing strategies to faces and found age-related perceptual changes specifically in integrating face features into global structures.

A number of studies have demonstrated that difficulties in face recognition with age might also reflect declines in high-level perceptual processes. For example, older adults have an impaired ability to (i) transform perceptual information into familiar templates that may explain poor recognition of faces when seen in other than the front view [13], (ii) engage in joint attention by following gaze cues [16], and (iii) ignore task-irrelevant information in faces [17].

There is also evidence that older people can experience difficulties in processing nonidentity related aspects of faces such as their emotional expressions [18–20]. For example, it has been demonstrated that older adults are less accurate in recognizing negative facial emotions, in particular, anger and sadness [19, 21], and they are less accurate in discriminating between some emotions (e.g., surprise and fear) [22], when compared with younger observers. The age-related decline in the processing of emotional expression has been linked to a variety of factors, for example, the tendency to establish a positive emotional preference in tasks as we age [23] and different perceptual demands for emotional processing relative to the coding of face identity, which may depend on cognitive abilities differentially affected by aging [24].

Given the wealth of studies suggesting age-related declines in both face recognition and the processing of emotional expression in faces in older individuals, it is surprising that no studies have attempted to investigate how aging affected the processing of combined information about facial identity and emotional expression, despite facial identities and expressions covarying in everyday life.

Interestingly there is evidence that the ability to integrate information from two signals is spared as we age [25–28] and older participants can even benefit more than younger adults when information from two sources is combined. For example, Laurienti et al. [27] examined the speed of older and young individuals to discriminate the presence of simple visual (a red or blue circle), auditory (the word red or blue), and combined visual-auditory stimuli. RTs were faster to targets with combined attributes and this “redundant target effect” was greater for older adults than for younger adults (see also [25, 26, 29]). These studies suggest that, although the processing of some properties of stimuli may decline in older individuals, there is a benefit when redundant signals are present to define targets. The enhancement in performance from redundant targets has been linked to the greater ability of older participants to exploit redundant information to help compensate for sensory deficits with single stimuli [27]. For example, if aging is associated with increased levels of “noise” in perceptual processing, then the presence of a redundant target may be beneficial if the additional signal information reduces the effects of the noise [29]. Hugenschmidt et al. [30] applied capacity analysis [31–34] to the data in Laurienti et al. [27]. This analysis compares reaction time (RT) distributions expressed through a hazard function [31] to estimate of how much “work” a system does and how much processing is influenced by stimulus manipulations in a task. The analysis indicated that older adults increased their capacity processing 2.5 times as much when redundant signals were present compared with single signals, while younger adults only increased their capacity up to 1.5 times. The mechanisms underlying such effects, however, remain poorly understood. The present study aims to address this last point by studying the dynamics of redundancy effects in face processing across the lifespan focusing on the relations between the processing of facial identity and emotion.

### 1.1. Processing Facial Identity and Emotion

A controversial issue in studies of face processing is whether facial identity and emotion are processed independently or in a more interactive manner. The influential model of face recognition proposed by Bruce and Young [35] suggests separate-parallel routes for processing identity and emotional expression. The main support for the model came from neuropsychological studies showing double dissociations of patients with impaired recognition of face identity but not emotion [36–39] or vice versa [36, 40]. Studies with healthy participants also provide some support for the parallel route hypothesis [41, 42].

On the other hand, there are a growing number of studies suggesting that the processing of identity and emotional expression in faces is not entirely independent [43–53]. For example, there is evidence that facial expressions have an influence on the perception of familiarity [45, 54] and that the presence of covarying expression improves the recognition of facial identity in patients [55].

Previously we have examined this issue about independent versus integrative processing of facial identity and emotion using a divided attention task where participants had to detect target identities or emotions in faces. The identity and emotion targets could either appear alone (e.g., the target identity might be paired with a neutral emotion) or appear together (the target identity also expressed the target emotion). Young adults showed a strong facilitation effect for faces containing both the target identity and emotion (a redundant target display) [56]. Moreover, tests were carried out to explore the underlying basis of the redundancy effect. In particular, we used the so-called Miller inequality test [57] to assess whether the gains when redundant targets were present were greater than the gains produced when performance with the two single targets was summed. Based on there being independent processing of facial identity and emotion, we would not predict that performance can be better than the summed responses to each target feature alone. The data violated this prediction, with the redundancy gains being greater than predicted from independent processing of each feature. Also by varying the features present on nontarget trials we also tested (and rejected) accounts of performance based on partial information from each feature (identity and emotion) influencing decision-making independently. We concluded that facial identity and emotion were processed in an integrated manner.

The present study is concerned with how the coding of facial identities and emotions is affected by age and, in addition to this, whether older participants, like younger individuals, process facial identity and emotional expression in an integral manner. To test this, we examined the detection of face identity and emotion targets in three groups of participants aged between 20 and 30, 40 and 50, and 60 and 70 years, with target faces carrying either the critical identity, the emotion, or both the identity and the emotion. We evaluated whether responses to redundant target faces were faster than responses to either single target and what is the magnitude of the redundancy effect in the different age groups examined.

### 1.2. Analyzing Redundancy Gains in Information Processing

There is considerable evidence that, when a visual display contains two targets that require the same response, reaction times (RTs) are faster than when only one target appears [57–60]. There are different explanations that account for this redundant target effect (RTE), with the most relevant being the independent race model [61] and the coactivation model [57]. The independent race model assumes separate and parallel channels for processing distinct types of information (e.g., facial identity and emotional expression) with the response to each trial determined by the fastest process to be completed. In a test using elementary visual targets, Miller [57] demonstrated that predictions made by the independent race model [61] were violated. The critical contrast for the two models compared the probability of responses being made within particular time bins on redundant targets trials relative to sum of the probabilities for responses in the same bins when either single target was present. The independent race model holds that at no point in the cumulative distribution functions for performance should a response to redundant targets exceed the sum of the probabilities for responses to either single target [57]. In contrast, if there is coactivation of a common representation of both features, responses to the redundant targets can be made from this common representation before processing is complete for either single target. The coactivation model put forward by Miller [57] closely resembles the capacity model of Townsend and Nozawa [62] which defines processing in terms of workload capacity [62]. The concept of capacity reflects the efficiency with which the cognitive system performs a task. In terms of this model, full parallel processing of stimuli (as maintained by the independent race model) is associated with unlimited capacity (*C*(*t*) = 1). Processing with limited capacity (*C*(*t*) < 1) is associated with decreasing performance (e.g., slowing in RT) when the workload increases. On the other hand super capacity (*C*(*t*) > 1)) is associated with integrated processing of features and this always violates the predictions made by the independent race model [31].

These models provide a formal framework within which we can explore the relations between the processing of facial identity and emotion in older adults. Here we tested whether redundancy gains in processing facial identity and emotion occur in older as well as young participants and whether the effects are linked to violations of the independent race model and to super capacity (*C*(*t*) > 1)) in information processing. These tests enable us to elucidate the mechanisms underlying face processing across the age range.

## 2. Method

### 2.1. Participants

Three groups of twelve healthy participants were recruited: (1) young (aged 20–30 y, 10 female), (2) middle-aged (aged 40–50 y, 8 female), and (3) elderly (60–70 y, 8 female). The young participants were University of Birmingham undergraduate students (second- and third-year students of Engineering Department); the middle-aged group was recruited from staff at the Queen Elizabeth Hospital in Birmingham (receptionists, statisticians, and nurses) and staff of University of Birmingham (receptionists and demonstrators); the older group consisted of individuals recruited to the School of Psychology's panel for older participants. There were no big variations between elderly and middle-aged participants in terms of educational level and positions held before their retirement. The majority of the older adults were office workers (secretary, administrator, public coordinator, receptionist, assistant in Post Office customer service, a clerical worker in Estate and Letting agent, rent officer, assistant of manager in a library, and a registrar at a job centre); two participants previously worked as school teachers. None of the volunteers had health-related issues or physical and mental disabilities. There was also a consistency across all participants in their experience of performing a face recognition task in an experimental situation; none of the participants has been previously recruited for a face recognition experiment.

All participants had normal or corrected-to-normal vision and this experiment was carried out in accordance with the ethical guidelines of the British Psychological Society. Each participant gave informed consent at the start.

### 2.2. Stimuli and Apparatus

**Figure 1:**
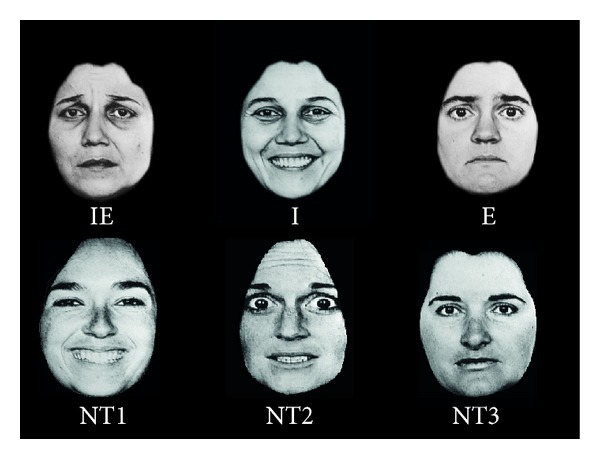
An example of the stimuli. IE: a face containing both the target identity and the target emotional expression; I: a face containing the target identity but not the expression; E: a face containing target emotional expression; NT1-NT3 faces containing neither the target identity nor the target emotion. In this study we used faces from the NimStim database, but, because of publication restriction on faces from that database, we present here other faces (taken from Ekman and Friesen [63]) as examples only.

A set of 6 photographs was employed (Figure 1).

All the face images were sourced from the NimStim Face Stimuli Set [64]. The recognition of facial expression in all the photographs was rated as 80% and higher [64]. The set consisted of white male faces (original images are not presented here due to restricted permission to publish images from the database).

The photographs were cropped around the hairline to eliminate the possibility of target judgments being based on hairstyle. Any visible background was coloured black. The faces were approximately 10 × 13 cm when displayed on a 17-inch monitor. The presentation of stimuli was controlled using Cogent 2000 and Cogent Graphics developed by the Cogent 2000 team at the FIL and the ICN (http://www.vislab.ucl.ac.uk/cogent_2000.php). The stimuli were presented on the monitor at the viewing distance of 0.8 m. The angular width subtended by the stimulus was approximately 10°.

### 2.3. Design and Procedure

A “go/no-go” task was employed. Half of the trials used stimuli containing at least one target attribute (target identity, target emotional expression, or both targets; “go” trials). On the other half of the trials, the stimuli did not convey any target attribute (“no-go” trials).

Participants were asked to respond as quickly and accurately as possible when the target identity and/or the emotional expression were displayed by pressing a button “target present” on the keyboard (the right arrow). To minimize bias found for processing positive emotions in older participants (e.g., [65]), the emotion target was a sad expression. The targets were Person 1 expressing a sad emotion (redundant targets), Person 1 with a happy expression (target identity and nontarget expression), and Person 2 with a sad expression (target expression and nontarget identity). The nontargets were a face of Person 3 with an angry expression (nontarget identity and nontarget emotion), Person 4 with a neutral expression (nontarget identity and nontarget emotion), and Person 5 with surprised expression (nontarget identity and nontarget emotional expression) (see examples of stimuli in Figure 1).

Prior to the main task participants completed an initial practice block of 18 trials during which they were given feedback on their accuracy and RT after each trial. After a short break participants performed four blocks of 60 trials with short breaks between the blocks. Each trial started with the presentation of a fixation cross at the centre of the screen for 500 ms. Images were presented successively in random order with random interstimulus intervals from 500 ms to 2 sec. On “go” trials the image was displayed until a response was made. On “no-go” trials the stimulus was displayed 2000 ms.

The procedure for the experiment was kept as consistent as possible across all participants (using the same testing room and computer, performing experiments in morning hours, etc.).

### 2.4. Analysis of Data

Four analyses were conducted in this study. The first looked at whether the three groups of participants differed in accuracy and RT performance across the different conditions. RTs for correct responses only were used in further analyses.

The second analysis determined whether participants were faster for redundant targets compare to single target trials. Mean RTs across the two single targets (e.g., emotion only or identity only) was subtracted from the mean RT for redundant targets for each participant. A positive value following this subtraction represents a redundancy gain. It has been shown that, when some observers favour one dimension over another, there is an overestimation of the mean RT redundancy gain relative to the fastest single dimension condition for each observer [66, 67]. In contrast, the fixed favored dimension test involves comparing the two single target conditions for each observer against each other. When the two conditions differ, the faster mean RT is retained as the conservative estimate of the single target mean RT; when the two conditions do not differ, the overall mean from both single target conditions is used.

The next analysis assessed whether the independent race model inequality was violated [57]. To test the Miller [57] inequality, empirical CDFs were estimated for every participant and every target condition. All calculations followed the algorithm for testing the independent race model inequality [68]. First, the 100 RTs generated by each participant for all target trials were sorted in ascending order to estimate 19 percentiles (5th through the 95th at 5% intervals). Subsequently these numbers were averaged across participants to produce the composite CDF for redundant targets and each single target condition. To produce the sum of CDFs for the target identity (I) and emotion (E) trials, RTs for the two types of trial were pooled together and 19 quintiles were estimated based on only the fastest 100 of the 200 trials. All calculations were conducted using a modified Matlab script for computing the independent race model test [68]. The nineteen percentiles points and CDFs were calculated for each participant and then averaged. Paired two-tailed* t*-tests were used to assess the reliability of the difference between redundant targets and the sum of target identity and target emotion responses at each percentile point. Graphic representations of the distributions were constructed using group RT distributions obtained by averaging individual RT distributions [68]. When the CDFs are plotted, the independent race model requires that the CDFs of the redundant targets fall below and to the right of the summed CDFs. Finally we examined whether the groups of young, middle-aged, and older participants were different in the magnitude of any redundancy gains.

The fourth analysis examined capacity processing. Here we used a method of computing the capacity coefficient proposed by Townsend and Eidels [31]:

(1) $$\begin{array}{c} C_{\text{OR}}\left(t\right)=\frac{-\log\left[S_{\text{IE}}\left(t\right)\right]}{-\log\left[S_{\text{I}}\left(t\right)*S_{\text{E}}\left(t\right)\right]}, \end{array}$$

where the survival function of the redundant targets condition is in the numerator and the product of the survival functions of the two single target conditions is in the denominator.

For this assessment, for each condition, we calculated the empirical CDFs using 10 ms time bins. After this the empirical survivor function was computed for each condition at each time bin, which is simply the complement of the cumulative distribution (the proportion of trials that was slower than the specified RT). All computations were performed using Matlab codes [31]. After averaging the CDFs for the redundant targets and either single face property (identity and emotion), the data were converted into survivor functions in order to create integrative hazard functions. Subsequently the capacity coefficients for each group of participants and each face set were generated by creating a ratio of the averaged hazard functions at each time bin [30]. Confidence intervals were defined for each group-capacity coefficient using the bootstrapping technique [31].

## 3. Results

### 3.1. Accuracy Performance

Performance accuracy across the three groups of participants is displayed in Table 1.

It is important to note that accuracy was very high across all groups, over 99% accuracy on “go” trials (target(s) present) and 96% on “no-go” trials (target absent). Group comparisons for accuracy performance were made using a 3 (groups) × 6 (conditions) repeated measures analysis of variance (ANOVA). There was a main effect of stimulus (*F*(5,165) = 11.3, *P* < 0.001, *Pμ* ^2^ = 0.025), in which participants made more errors on “no-go” than on “go” trials (Table 1); a main effect of group (*F*(2,33) = 4.0, *P* < 0.05, *Pμ* ^2^ = 0.02), in which the older the participants were, the more errors they made; and an interaction of stimuli ∗ group (*F*(10,165) = 3.3, *P* = 0.001, *Pμ* ^2^ = 0.017). Adjustment for multiple comparisons (Bonferroni) revealed that the interaction was mostly driven by the responses to the “no-go” trials with the young participants being more accurate in responding to nontarget stimuli (see Table 1) as compared to the older participants (*P* < 0.05). There were no reliable differences in the accuracy of performance between young and middle-aged participants or between middle-aged and older groups (all *P* > 0.05).

### 3.2. RT Performance

**Figure 2:**
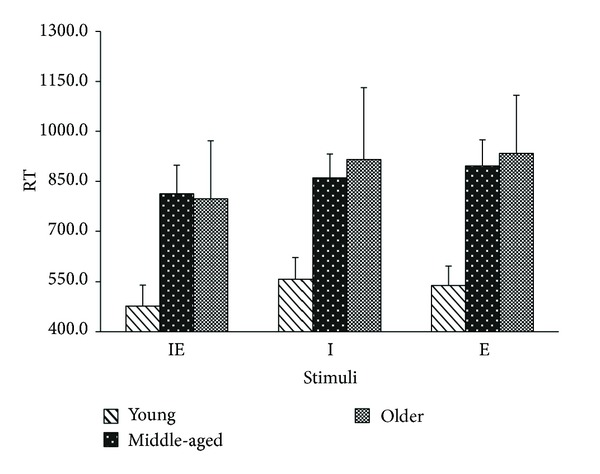
Mean RTs (SD) for images containing both targets (IE), the identity target (I), and the emotional expression target (E), for groups of young, middle-aged, and older participants.

Mean RTs and standard deviation for correct responses to stimuli containing targets for three groups of participants are displayed in Figure 2.

There was no evidence for accuracy/RT tradeoffs (see Supplementary Material 1 Available online at http://dx.doi.org/10.1155/2014/136073). Group comparisons for RT performance were carried out using a 3 (conditions containing targets) × 3 (groups) ANOVA. As expected, age group had a reliable effect on response times (*F*(2,33) = 33.13, *P* < 0.001, *Pμ* ^2^ = 0.066), with younger participants being faster than both the middle-aged and older groups on all conditions (all* t*(22) > 5.5, *Ps* < 0.001, *ds* > 0.52), though the middle-aged and the older groups showed no reliable RT differences (*Ps* > 0.05). Target type also had an effect on RT (*F*(2,64) = 32.8, *P* < 0.001, *Pμ* ^2^ = 0.06), such that RTs on redundant target (IE) trials were faster than both of the single target conditions (IE versus I:* t*(35) = 9.3, *P* < 0.001, *d* = 0.96; IE versus E:* t*(35) = 10.8, *P* < 0.001, *d* = 0.97). This effect was reliable in all three groups: (IE versus I + E: young, middle-aged, and elderly).

More interestingly we also observed an interaction between age group and stimuli (*F*(4,64) = 5.4, *P* = 0.001, *Pμ* ^2^ = 0.021). Inspection of Figure 2 suggests that the redundancy effect increased with age.

To ensure that our results were not caused by general age-related slowing, which might produce spurious interactions between age group and the experimental conditions [69], we also examined the effects after we converted each individual's RTs to log-transformed scores. Using the log transformation here instead of commonly used *z*-score transformation [69] was driven by our data which showed correlations for all conditions across the three participant groups (see Supplementary Material 2) and high individual variability in responses to the same stimuli (Supplementary Material 3) that might bias against using *z*-score transformations. Salthouse [70] suggested that an interaction of Task × Age that remains statistically significant after log transformation of raw RTs is the result of age effects beyond more general influences of task complexity and general slowing, because a logarithmic transformation represents equal ratios as equal intervals. The new transformed scores were then used in a mixed design ANOVA with targets (IE, I, and E) as within-subject factor and age group (young, middle-aged, and older) as a between-subject factor. This analysis revealed a very similar result to the analysis of the nontransformed data; there was a main effect of group (*F*(2,32) = 43.8, *P* < 0.001, *Pμ* ^2^ = 0.068) and of stimulus (*F*(2,64) = 43.7, *P* < 0.001, *Pμ* ^2^ = 0.05) and an interaction of stimuli∗group (*F*(4,64) = 5.6, *P* = 0.001, *Pμ* ^2^ = 0.021). There were no other effects (*F* < 1.0). Similar to the ANOVA on the nontransformed data, patterns for the three stimuli were obtained after the examination of main effects using independent sample* t*-tests.

### 3.3. Redundancy Gains

All three groups showed faster responses for displays containing both targets compared to displays containing either single target (Figure 1). To better understand this effect we computed the redundancy gain for each participant, by subtracting the RT of the redundant target from each single target, separately. The redundancy gain was then estimated conservatively based on the smallest of the two subtractions. These data were entered into a one-way ANOVA performed on nontransformed data which were used to test for the size of redundancy gains (RGs) among young, middle-aged, and older participants.

**Figure 3:**
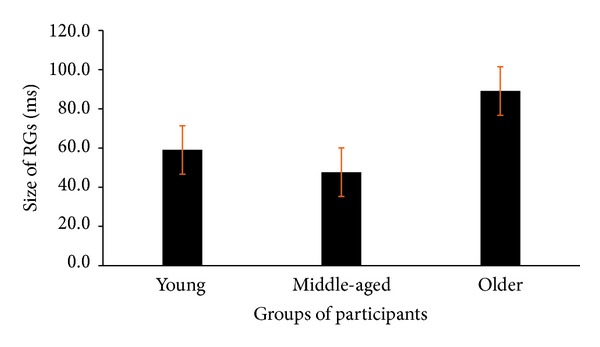
The size of the redundancy gains for groups of young, middle-aged, and older participants.

In line with the interaction that we reported above, the size of the RGs was reliably different across the participant groups (*F*(2,33) = 5.1, *P* = 0.012, *Pμ* ^2^ = 0.24). The redundancy gain was larger for the older participants as compared to the middle-aged group (*t*(22) = 2.8, *P* < 0.05, *d* = 0.75) (Figure 3). The differences between the younger and the older groups, and between the younger and the middle-aged groups, were not reliable (all *Ps* > 0.05).

The same analysis using log-transformed data revealed quite similar results for the main effect of group (*F*(2,33) = 5.35, *P* = 0.01, *Pμ* ^2^ = 0.27). However, post hoc (Bonferroni) comparisons showed that, in contrast to the nontransformed data, the size of the redundancy gain in both younger and older participants was reliably greater than in the middle-aged group (*t*(22) = 3.0, *P* = 0.048, *d* = 0.52, and *t*(22) = 2.7, *P* < 0.05, *d* = 0.74, resp.). Similar to nontransformed data no difference in the size of the redundancy gain was found for comparisons between young and older participants (*t*(22) = 0.4).

### 3.4. Testing the Independence versus Coactivation

To test whether redundancy gains are determined by statistical sampling of independent channels or coactive processing of identity and emotional expression, we carried out the Miller test [57] for each RT quantile point in the three groups using paired-sample* t*-tests (comparing mean RTs for the redundant targets to the sum of the identity target and the emotion targets, at each quantile point in each group of participants). As mentioned in the data analysis section, Miller's inequality [57] predicts that, for the independent race model [61] model, the probability of a response in the redundant target condition should never exceed that for the sum of two single targets [57]. Previous studies have reported violations of the Miller inequality [57] for early percentile points (e.g., percentiles 10–25; [26, 58]). In cases where violations of the inequality are observed at multiple percentile points, an appropriate correction for multiple comparisons is needed in order to control Type 1 errors. However, the widely used Bonferroni test for multiple comparisons demands that all the tests be independent of each other [71]. This demand is not fulfilled in our data where correlations between percentiles bins are high (from 0.84 to 0.93). In order to conduct the Miller test [57], but to control Type 1 errors, our strategy was to perform the test at two percentiles (10% and 15%) using paired* t*-tests but with a strict significance level adopted (1% instead of 5%).

**Figure 4:**
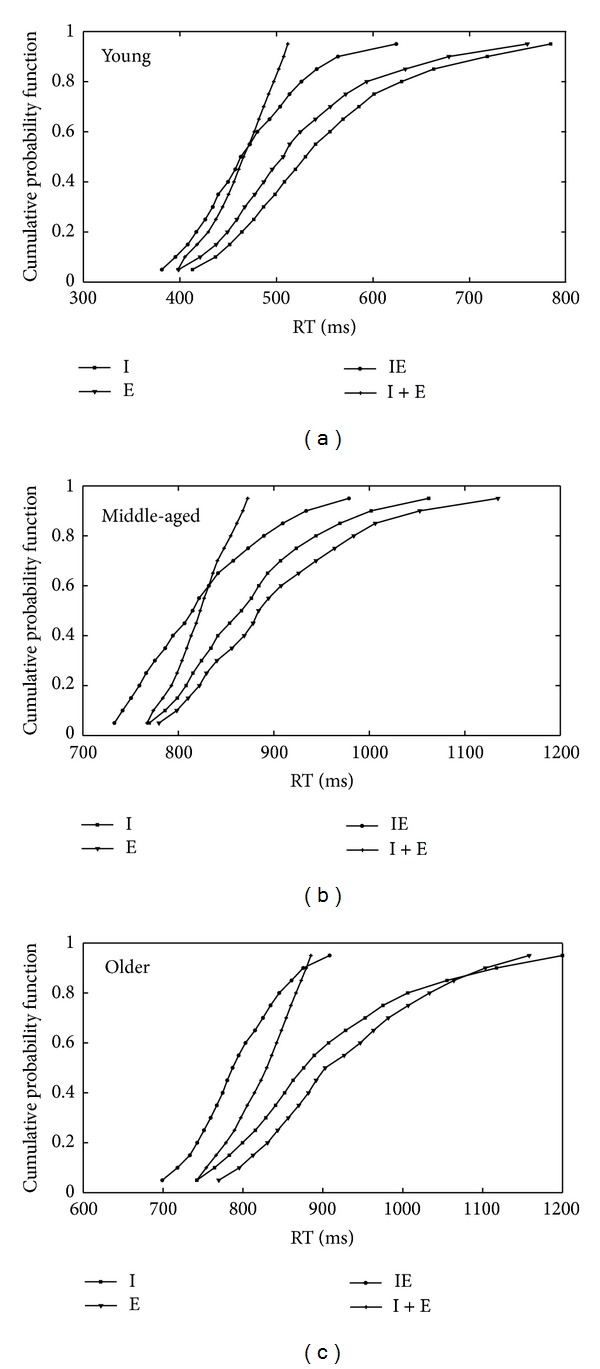
CDFs for redundant targets (IE), the sum of the distributions for emotional expression and identity targets (I + E), and single targets (E) and (I) in groups of young (a), middle-aged (b), and older (c) participants.

The CDFs for each participant group are displayed in Figure 4. The CDFs for redundant targets fell to the left of the sum of the single target CDFs for the redundant target faces, which indicates violation of the Miller [57] inequality. These violations were statistically significant at the 10th and 15th quantiles (*Ps* < 0.01) in all groups of participants.

**Figure 5:**
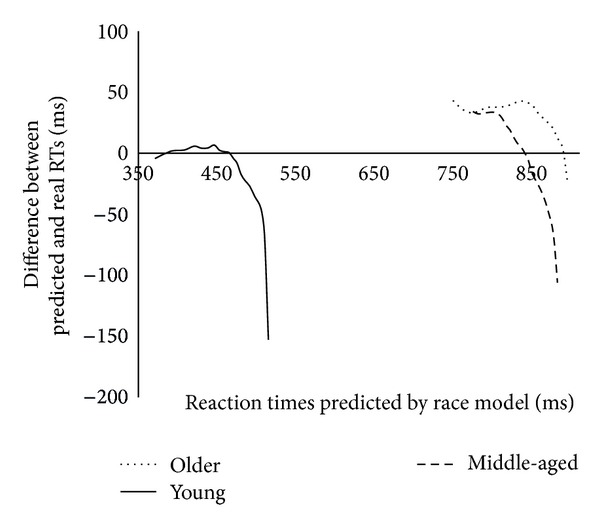
Differences between RTs predicted by the race model (solid horizontal line) and the observed RTs for young, middle-aged, and older groups.

To visualize the results above, we plotted the CDFs for each quantile point predicted by the race model (I + E) from the CDFs of corresponding quantiles for redundant targets (IE) (see Figure 5).

The data presented in Figure 5 demonstrate that the time window over which the older group benefitted from redundant targets was larger than that in the young and the middle-aged groups (the part of the curves that are above the horizontal solid line): for the young group the time window for the violation ran across 50 ms (440–390 ms), for the middle-age participants it was 75 ms (840–765 ms), and for the older group it was 201 ms (900–699 ms). Also, the magnitude of the enhancement from redundant targets was reliably greater for the older group (*F*(2,33) = 8.92, *P* < 0.05, *Pμ* ^2^ = 0.053) than for the young group (*t*(22) = 4.2, *P* < 0.05, *d* = 0.54) and the middle-aged group (*t* (22) = 2.8, *P* = 0.045, *d* = 0.21). The young and middle-aged groups did not differ.

### 3.5. Testing Capacity Processing

**Figure 6:**
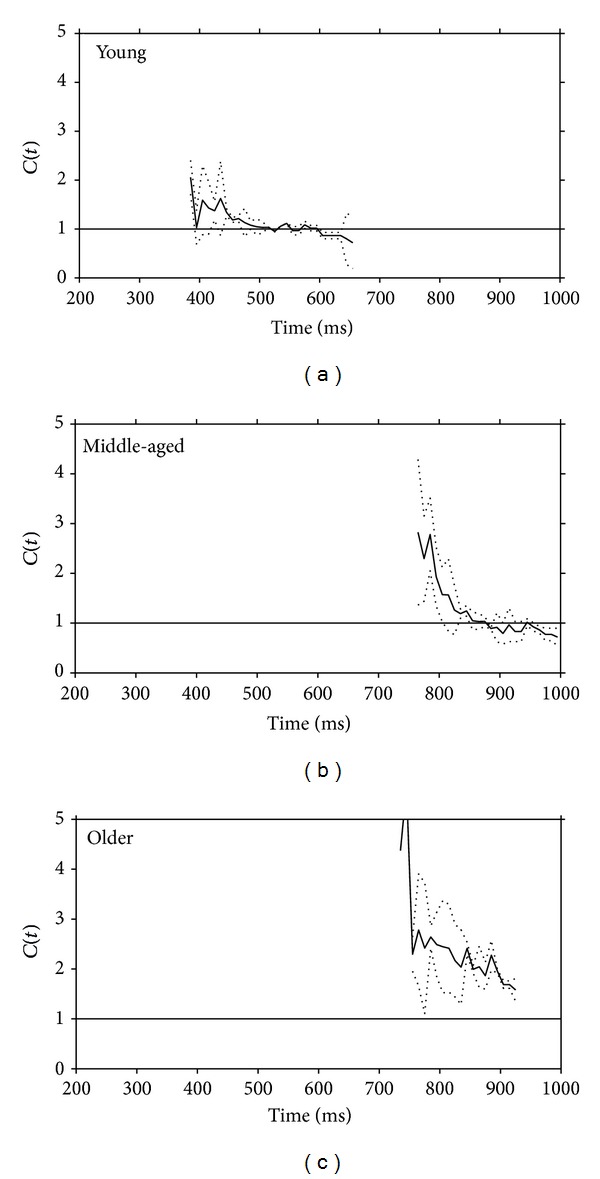
Capacity coefficients for the three groups of participants: top row young adult and middle row middle-aged people. The horizontal line at *C*(*t*) = 1 indicates the reference value for unlimited capacity. The capacity coefficients are depicted in solid line; the confident interval for capacity coefficient is depicted in dash line.

The capacity coefficients at each time bin across participant groups are presented in Figure 6, and the overall capacity coefficients (averaged across time bins) are displayed in Table 2.

Figure 6 and Table 2 demonstrate different magnitudes of capacity processing in the three groups of participants. The overall workload capacity in the older group was significantly greater in the redundant target condition (*F*(1,33) = 8.9, *P* < 0.001, *Pμ* ^2^ = 0.49) when compared to the young participants (*t*(11) = 5.18, *P* < 0.001, *d* = 0.41) and the middle-aged group *t*(11) = 4.6, *P* < 0.001, *d* = 0.62). The difference between the younger and middle-aged groups was not reliable (*t*(11) = 0.8).

## 4. Discussion

The present study examined the effect of aging on the processing of redundant information in faces. Three findings are striking. First, participants from all three age groups benefitted from combining facial identity and emotional expressions in target faces, but this effect was more pronounced in the older group which showed greater redundancy gains in processing both the identity and emotional expression targets. Second, the results of the Miller test [57] provide clear evidence that enhanced responses to redundant face stimuli are determined by coactive processing of the two facial dimensions (note that Yankouskaya et al. [56] provided evidence against redundancy gains reflecting crosstalk after partial processing of facial identity and emotion.). The coactive processing was present at all ages but had more marked effects on the performance of the older participants. Third, the capacity analysis indicates that older people were able to increase their processing up to 2.4 times with redundant targets, while younger and middle-aged adults increased their capacity only 1.2 times. Most notably, the effects of age here remained even when the data were log transformed, when effects of overall processing speed should be reduced [6, 72, 73].

These findings are consistent with the results of previous studies with elementary visual stimuli showing faster responding for two targets as compared to either single target in elderly participants [25–27, 74] and super capacity in the processing of multisensory stimuli [30]. The new findings here are as follows: (1) the redundant target effect in older people occurs with complex ecological stimuli such as human faces; (2) facial identity and emotional expression are processed interactively, and this effect is preserved across lifespan; (3) the dynamics of the redundancy effect across lifespan have a nonlinear trend.

The results raise the question as to why older adults show improvements in processing of redundant information in faces, despite suffering age-related perceptual and cognitive declines, and what is the origin of this improvement? One possibility is that older people combine information from faces in a more efficient way compared to young adults due to life experience with faces. Indeed, there is evidence that accumulated life and interpersonal experience increases older adults' ability to identify other people's faces and emotions [75, 76]. As a result, this would predict an improved ability to combine information in faces and greater redundancy gains compared to younger adults. On the other hand, older subjects may process facial features less efficiently than younger adults, and the gains produced from redundant target faces are greater for individual features which are processed slower. This explanation is supported by previous studies demonstrating that older people showed increases in scanning behavior when looking at faces and were subsequently less efficient in recognition performance due to disrupted feature processing. There is also the possibility that greater redundancy gains in the older group may reflect a decline in ignoring irrelevant information accompanied either single target. For example, Allen et al. [77] emphasized that older participants have difficulties with ignoring irrelevant information rather than any difficulty associated with activating or selecting targets. If age increases the “internal noise” in information processing [77], then we can think that redundant targets contain no internal noise, while the single target stimuli contain “noise” along the other dimension (the “noise” of the emotional expression for identity targets and of identity information for expression targets). The failure to ignore stimuli along the nontarget dimension when only one facial feature is present would then lead to noise entering any computations on single target trials but also large(r) reductions in noise when both identity and emotion targets are present.

It has to be noted that although all the above explanations may account for the greater redundancy gains in the older group, it is less clear why the older group shows a greater integrative effect and higher workload capacity than younger adults, in the formal analyses presented here. The most pronounced evidence for enhanced integrative processing in elderly participants comes from studies using multisensory signals [25, 27, 74]. For example, similar to our results, Laurienti et al. [27] reported a greater peak and a broader temporal window of audio-visual integration. The authors suggest that the multisensory signals become much more efficient for older participants because these individuals experience declines in each of the unisensory modalities. Furthermore, an additional possibility that may account for the multisensory enhancement was linked to a better ability in the elderly to exploit the redundant nature of the cues. Bucur et al. [25, 26] proposed that older people do exhibit age-related deficits when they divide their attention between two different stimulus modalities; however, this is not sufficient to prevent coactivation from occurring.

Although the mechanisms of the enhanced responses for redundant information remain unknown, the evidence that, compared to younger adults, older people can better exploit redundant cues may explain the greater coactivation in processing both the identity and emotional expression targets. Given the evidence that older adults experience difficulty in integrating individual facial features into a whole representation, capturing covariations in redundant faces may be an efficient compensation strategy to minimize declines in the processing of facial identity and emotional expression. This assumption is supported by results of the capacity analysis here. The finding that the older group was generally slower but greater in processing capacity compared to the young group suggests that there is a qualitative difference in face processing across the groups. This difference may reflect some age-related changes in perceptual mechanisms. We can speculate here that combining target information from two sources may produce “a distinct feature” that makes the face containing both the identity and emotional expressing targets more salient. Responding to the “distinct feature” determines greater redundancy gains as compared with target information in either single target face, because this would require less cognitive resources. The challenge for further research is to examine whether reducing cognitive load by efficient integration information is a function of normal aging.

The data here show that the size of the redundancy gains in the middle-aged group was smaller compared to both the younger and older adults. This indicates nonlinear changes in face processing as people age. As compared to younger adults, middle-aged people experience some declines in face processing that results in smaller size of redundancy gains. Indeed, there is evidence that peak recognition performance for faces occurs around 30–34 years, reflecting the continued development of face processing skills into middle-aged people (Germine, Duchaine, and Nakayama, 2010). Smaller redundancy gains in the middle-aged group as compared to older participants suggest less efficient combining information and indicate that qualitative changes in perceptual mechanisms link to more advanced age. On the other hand, our results on the Miller [57] test of the independent race model, along with the capacity analysis, demonstrate gradual increases in coactivation and capacity processing across the groups. Although the finding needs further investigation, this may indicate that the integration of facial identity and emotional expression information is a function of normal aging.

A number of studies have reported faster RTs and higher accuracy for older participants when recognizing positive as compared to negative emotional expressions [78–80]. In the present study, the identity target only (Person 1) was shown with a happy emotional expression (nontarget emotion), while both the redundant target (Person 1 expressing sadness) and the emotional expression target only (Person 2 expressing sadness) contained a negative emotional expression. The “positive expression bias” predicts that the older group would show better performance for the identity target as compared to the emotional expression target, and there should also be a reduced redundant target effect. However, there were no reliable differences between RTs for the identity and emotional expression targets and an increased redundancy effect, contradicting the “positive emotion” bias account of performance. We did, however, find that the size of redundancy gain in middle-age participants was smaller as compared to both the young and the older groups when the log-transformed data were considered. Although the difference between RTs predicted by the race model and the observed RTs gradually increased with age (Figure 5), this finding needs further investigation to examine which factors may reduce redundancy gains in middle age.

## 5. Conclusions

The present study provides strong evidence that facial identity and emotional expression are processed interactively and this interaction remains intact with age, with, if anything, older people demonstrating a greater benefit when identity and emotion combine in a redundant manner. This effect does not reflect general age-related slowing but may result from increased distribution of attention to both identity and expression properties of faces in older participants.

## Supplementary Material

It was found that correlation between response latencies for each stimulus on “go” trials and number of errors was small and not reliable, excluding correlation for the target identity in the older group. The elderly showed significant positive correlation between RT and errors for the stimulus containing target identity. Given that a difference in RT between the identity and emotion targets was not significant and the positive character of the correlation did not imply for speed-accuracy tradeoff, the accuracy performance could be ignored in redundancy gain analyses for the older group.

## Figures and Tables

**Table 1:** The mean percentage of errors for redundant targets (IE), the identity target (I), the emotional expression target (E), and the 3 nontarget faces (NTs) for groups of young, middle-aged, and older participants.

Group of participants	Stimuli					
	IE	I	E	NT1	NT2	NT3
Young	0.2	0.3	0.3	0.6	0.5	0.5
Middle-aged	0.1	0.2	0.4	0.3	1.3	1.6
Older	0	0.4	0.6	0.3	3.4	2.2

Total	0.3	0.9	1.3	1.2	5.2	4.3

**Table 2:** Overall capacity coefficients for young, middle-aged, and older people.

Capacity coefficient	Groups		
	Young	Middle-aged	Older
	1.12	1.24	2.24
