# Supplementary material for: Interactions between Identity and Emotional Expression in Face Processing across the Lifespan: Evidence from Redundancy Gains
Source: J Aging Res. 2014 Apr 15;2014:136073. doi: 10.1155/2014/136073 (PMC4009258; doi:10.1155/2014/136073)
Supplement: Supplementary file 1 — It was found that correlation between response latencies for each stimulus on “go” trials and number of errors was small and not reliable, excluding correlation for the target identity in the older group. The elderly showed significant positive correlation between RT and errors for the stimulus containing target identity. Given that a difference in RT between the identity and emotion targets was not significant and the positive character of the correlation did not imply for speed-accuracy tradeoff, the accuracy performance could be ignored in redundancy gain analyses for the older group. [file 136073.f1.pdf]

Supplementary material

1. Examining accuracy/RT trade off

The overall correlation between RT and accuracy in the three groups of participants is displayed in Table A.

Table A. *The overall correlation between RT and number of errors for the redundant targets (IE), the identity target (I), and the emotional expression target (E) in groups of young, middle-aged and older participants*

Groups of participants	Stimuli		
	IE	I	E
Young	0.31	-0.42	-0.16
	(p> .05)	(p> .05)	p> .05)
Middle-aged	0.38	0.1	-0.17
	(p> .05)	(p> .05)	(p> .05)
Older	*	0.64	0.08
		(p< .05)	(p> .05)

- the value was not computed because of 100 % accuracy

Correlation between response latencies for each stimulus on “go” trials and number of errors was small and not reliable, excluding correlation for the target identity in the older group. The elderly shows significant positive correlation between RT and errors for the stimulus containing target identity. Given that a difference in RT between the identity and emotion targets was not significant and the positive character of the correlation does imply for speed-accuracy tradeoff, the accuracy performance can be ignored in redundancy gain analyses for the older group.

2. Examining a proportional relation in RT performance for each condition containing targets across young, middle-aged and older groups

To assess whether the age differences in speed variables are attributable to the operation of a single slowing factor or reflect specific (or at least non-general) effect, systematic relation between stimuli were defined using Brinley plot. Brinley plot is a graphical representation of the proportional relation in RTs for a task conditions across groups (i.e., if older adults are slower than younger adults by an amount proportional to the RTs of younger subjects, then Brinley plot will represent linear relation between performance in the older and young groups). Figure A demonstrates such relation between groups of participants.

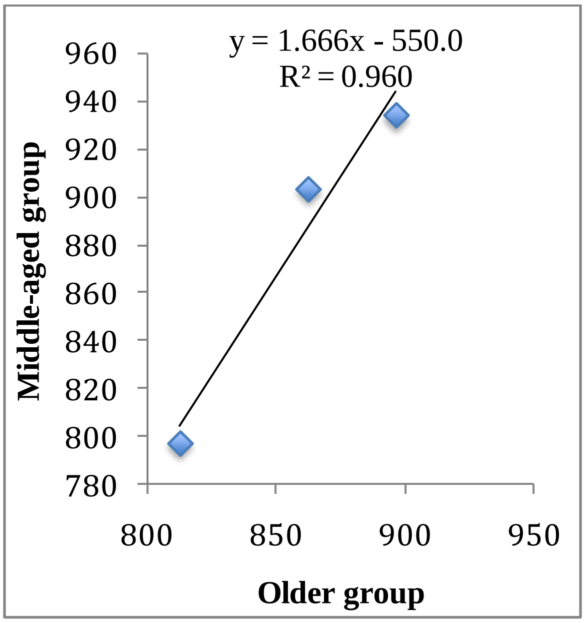

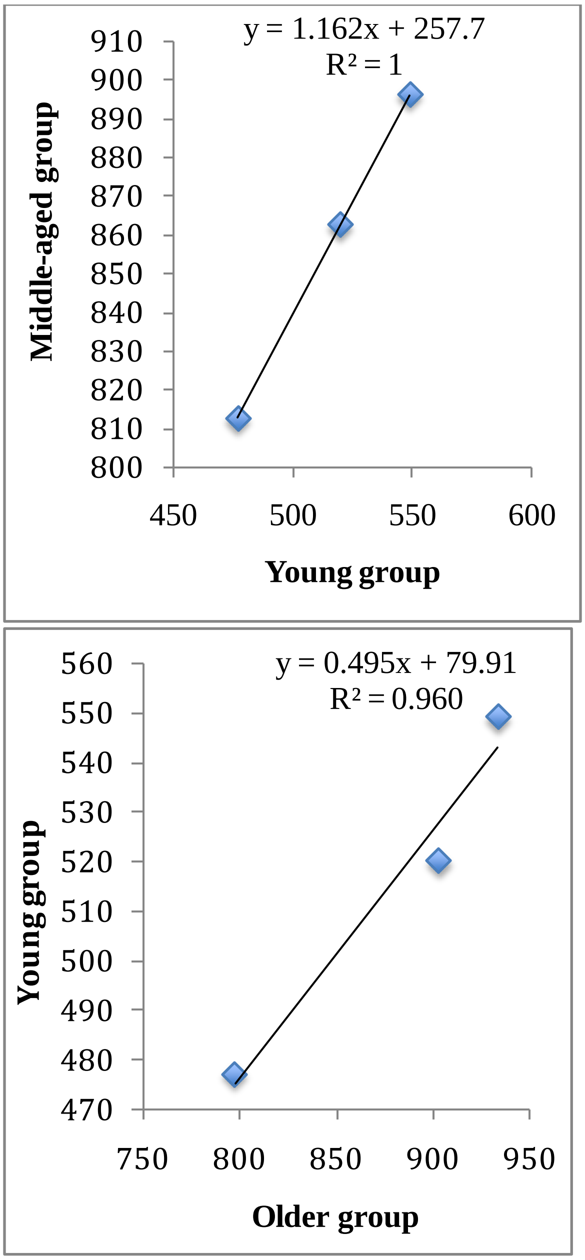

Figure A. Systematic relation between RTs for the redundant target, the identity target and the emotion target (three blue dots) for groups of young, middle-aged and older participants. Each point in each scatterplot corresponds to mean RT for a particular target, with the abscissa representing RTs (ms) for one group, and the ordinate representing RTs (ms) for the other group. The solid line is the regression line relating the values for the two particular groups in each scatterplot.

Figure A illustrates relations when the mean response times of older versus

young, young versus middle-aged and middle-aged versus older subjects are plotted for each condition containing targets. The slops and intercepts displayed in Figure A are in a line with previous studies reported a slope greater 1 and intercepts varied between +214 ms and - 693 ms (Cerella, 1991; Faust, 1999). Interestingly in each case the slope and intercept are different. In the present experiment participants' performed target detection task on 6 conditions, three of them required response 'target present'. If the difference between groups in responding for stimuli containing targets was caused by age-related slowing then we should expect similar parameters for the Brinley plots across all groups. One explanation of this result may be that relative placements of the means and standard deviations of RT distributions for each group different (Ratcliff, Spieler&McKoon, 2000). For example, Ratcliff et al. (2000) using the relative standard deviations of older versus young subjects' response times showed that the linear relation arose from a larger spread in the distribution of mean response times for older subjects than for young subjects. For instance, the negative intercept of the Brinley plot was linked to larger older subjects' means than younger subjects' means, but not by too much, relative to their standard deviations. Further, Ratcliff at al. (2000) demonstrated that difference in the patterns of RT distributions in older and young groups might be due by different response criteria, different quality of information that older subjects

3. Individual variability in responding for stimuli containing targets across young, middle-aged and older groups

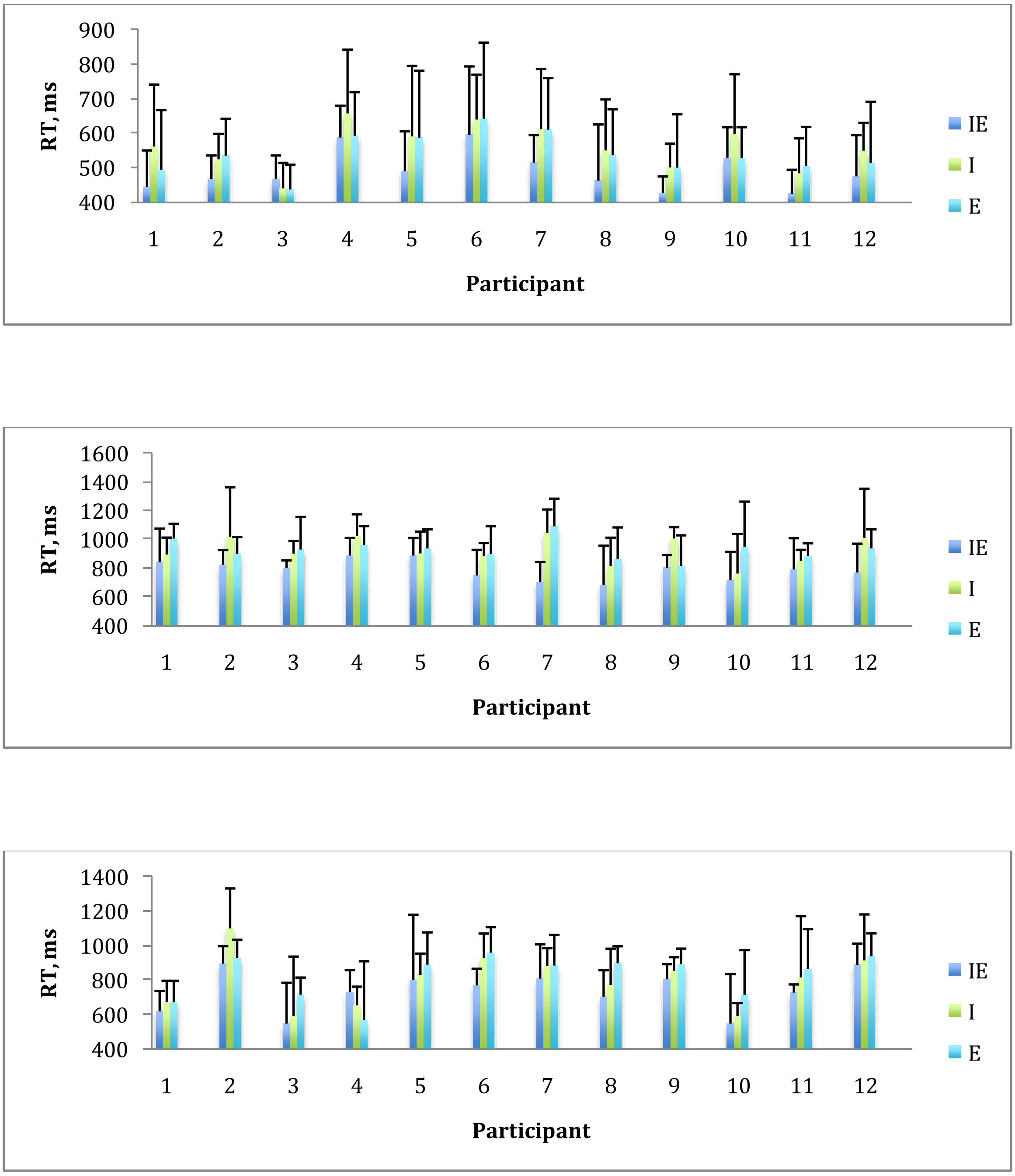

Figure A. Mean RTs for the redundant targets (IE), the identity target (I), the emotional expression target (E) in group of young (top), middle-aged (middle) and older people
